# Intraprocedural atrial tachycardia during ablation of paroxysmal atrial fibrillation: incidence, mechanisms, and clinical outcomes

**DOI:** 10.3389/fcvm.2026.1822766

**Published:** 2026-04-22

**Authors:** Qiushi Chen, Youfu Huang, Yan Dong, Xuesheng Fan, Nishant Yadav, Li Jiang, Yuan He, Zhongda Chen, Wei Zhao, Fengxiang Zhang

**Affiliations:** Section of Pacing and Electrophysiology, The First Affiliated Hospital of Nanjing Medical University, Nanjing, China

**Keywords:** CPVI, electrophysiological features, intra-procedural atrial tachycardia, paroxysmal atrial fibrillation, radiofrequency ablation

## Abstract

**Background:**

Radiofrequency catheter ablation (RFCA) is the mainstay treatment for paroxysmal atrial fibrillation (PAF). However, the incidence, risk factors, and impact of intra-procedural atrial tachycardia (IAT) during PAF ablation remain insufficiently characterized. The purpose of this study was to explore the incidence, risk factors, electrophysiological features, and clinical outcomes of IAT.

**Methods:**

In this single-center, prospective study, 255 patients undergoing RFCA for PAF were analyzed. IAT was defined as stable tachycardia lasting more than 2 min, either induced or spontaneous. Logistic regression identified risk factors for AT, and Kaplan–Meier analysis was used to examine its impact on long-term success.

**Results:**

IAT occurred in 13.33% of patients. Right atrial enlargement was identified as an independent risk factor [odds ratio (OR) = 1.14, *P* = 0.015], and AT involving the peri-tricuspid (45%), peri-mitral (45%), roof-dependent (2.5%) and focal (7.5%) types were found. The overall 12-month sinus rhythm maintenance rate was 78.0%, with no significant difference between the AT and non-AT groups (79.4% vs. 77.8%, *P* = 0.84).

**Conclusions:**

IAT was observed in 13.3% of patients undergoing PAF ablation, with macro-reentrant circuits around the tricuspid and mitral annuli being the primary mechanisms. Right atrial diameter served as a key predictor. Our data demonstrate that with successful intra-procedural identification and targeted ablation, IAT patients can achieve a 12-month prognosis similar to non-IAT patients.

## 1 Introduction

Radiofrequency catheter ablation (RFCA) stands as the mainstay and effective treatment modality for atrial fibrillation (AF) (1, 2). A cornerstone of RFCA for AF is circumferential pulmonary vein isolation (CPVI), given that ectopic triggers originating from the pulmonary vein (PV) sleeves are significant in initiating AF episodes (3). While CPVI is broadly performed, ablation strategies can vary, particularly for non-paroxysmal AF, leading to diverse incidences of post-procedural atrial tachycardia (AT). Specifically, ablation targeting fractionated potentials and the failure to achieve bidirectional conduction block during linear ablation have been linked to an increased risk of iatrogenic AT following AF ablation (4, 5). The occurrence of intra-procedural atrial tachycardia (IAT) during paroxysmal AF (PAF) ablation necessitates further elucidation, including its associated risk factors, common subtypes, and its effect on one-year post-ablation outcome.

## 2 Materials and methods

### 2.1 Study design and participants

This was a single-center, prospective, observational study. It included all consecutive patients undergoing PAF ablation at the First Affiliated Hospital of Nanjing Medical University between January 2022 and December 2023. The indication for RFCA was symptomatic PAF. The inclusion criteria for IAT was the occurrence of intra-procedural stable AT, either induced or occurring spontaneously, and persisting for ≥2 min (6). This duration was selected to distinguish clinically relevant, stable arrhythmias from transient, non-sustained atrial ectopy or organized triggers commonly observed during the ablation.

Exclusion criteria comprised: 1) Severe valvular heart disease, 2) Significant structural heart disease, 3) Prior cardiac surgery or structural interventions, 4) Presence of left atrial thrombus, 5) Recent surgical procedures within the preceding three months, 6) Severe hepatic or renal dysfunction (AST or ALT > 3 times upper limit of normal; serum creatinine > 3.5 mg/dL or creatinine clearance < 30 mL/min), 7) Allergy to low molecular weight heparin, warfarin, or novel oral anticoagulants (NOACs), 8) Life expectancy < 1 year, 9) Pregnancy or lactation, 10) Pre-procedural AT diagnosis, 11) Left atrial diameter ≥ 55 mm, 12) Platelet count < 80 × 10⁹/L, 13) Hyperthyroidism. Written informed consent was obtained from all patients prior to the procedure. The institutional ethics review board approved the study protocol (Approval NO. 2023-SR-895).

### 2.2 Pre-procedural preparation and research materials

To ensure procedural safety and establish baseline cardiac dimensions, contrast-enhanced ECG-gated cardiac CT or TEE was performed within 24 h before ablation to rule out intra-cardiac thrombi. Transthoracic echocardiography (TTE) was subsequently conducted for all the enrolled patients. Right atrial diameter (RAD) was measured in the apical four-chamber view at end-systole, while left atrial diameter (LAD) was measured in the parasternal long-axis view at end-systole using M-mode echocardiography. For all the patients on novel oral anticoagulants or warfarin, the international normalized ratio (INR) was maintained in between 2 and 3 for more than 3 weeks. Amiodarone was discontinued at least 4 weeks prior to the ablation, and other antiarrhythmic medications were stopped for at least 5 half-lives prior to the procedure. Patients were kept nil per os (NPO) for at least eight hours before the procedure, and RFCA was performed under local anesthesia and analgesic. All clinical and procedural patient data, including age, sex, body mass index, comorbidities, medication history, types of anticoagulants used, echocardiography findings, and intra-procedural parameters, were retrieved from the medical record system and intraprocedural records.

### 2.3 Electrophysiological study, mapping strategies and ablation

#### 2.3.1 Procedural settings and initial ablation

All procedures were performed using the CARTO 3 system (Biosense Webster, CA USA). High-density mapping was conducted with a Pentaray catheter with 4-4-4 mm spacing (Biosense Webster), and ablation was performed using a Thermocool SmartTouch ST/SF catheter (Biosense Webster). The primary strategy for PAF was circumferential pulmonary vein isolation (CPVI). Ablation parameters were set at a contact force of 15 ± 5 g, power of 35–45W, and an ablation index (AI) of 350–500. For substrate modification in low-voltage areas (LVAs) or coronary sinus ablation (20–25W), techniques followed previously established protocols (7, 8).

#### 2.3.2 IAT induction and provocation

Following CPVI, induction of IAT was systematically attempted via programmed atrial stimulation (PAS) from the high right atrium or proximal coronary sinus. PAS included extra-stimuli (S2/S3) and rapid burst pacing (200 ms CL) at twice the diastolic threshold. If no stable IAT (defined as a consistent activation sequence and CL lasting >2 min(6) was induced at baseline, isoproterenol (2–5 µg/min) and adenosine triphosphate (ATP, 20–40 mg) were administered to unmask dormant PV potentials or non-PV triggers. Notably, superior vena cava isolation (SVCI) was performed only when pathological triggers or rapid firing were documented and longer SVC myocardial sleeve length was found during mapping.

#### 2.3.3 IAT mapping and mechanism identification

Once a sustained IAT was found, high-density mapping was performed using the Coherent Module. Mapping points were automatically acquired based on strict stability criteria: TCL variation <10 ms, position displacement <3 mm, LAT difference <3 ms, and a point density of 1 mm. The mechanism of IAT was classified as follows:(1) Macro-reentrant AT: Confirmed by a “head-to-tail” activation pattern covering ≥90% of the TCL. Specifically, peri-mitral, peri-tricuspid, and roof-dependent flutters were diagnosed via activation sequences and confirmed by entrainment mapping (PPI-TCL <30 ms at critical isthmuses). (2) Focal AT: Identified by centrifugal spread from the earliest activation site, a unipolar “QS” pattern at the origin, and prompt termination during localized radiofrequency delivery.

#### 2.3.4 Ablation endpoints

For macro-reentrant IAT, linear ablation was performed to achieve bidirectional conduction block (9–11). Throughout the procedure, the activated clotting time (ACT) was maintained at 300–350 s.

### 2.4 Follow-up

Following the procedure, all patients were prescribed propafenone and oral anticoagulants during the 3-month blanking period. Antiarrhythmic drugs were routinely discontinued after this period unless recurrent arrhythmias were documented. Clinical success was defined as the absence of any atrial tachyarrhythmia (AF/AT/AFL) lasting >30 s off-AADs after the blanking period. Subsequent continuation of these medications was based on arrhythmia recurrence and symptoms. Before discharge, 12-lead electrocardiogram (ECG)was done in all the patients. 24 h ambulatory Holter monitoring was performed at 1st, 3rd, 6th, and 12th month post-procedural follow-up. A 12-lead ECG would be done at any time on presentation with symptoms of recurrence. Atrial arrhythmia recurrence was defined as documented episodes of AF, AT, and/or atrial flutter persisting >30 s, recorded on ECG or Holter monitoring after the 3-month blanking period post-ablation.

### 2.5 Statistical analyses

Continuous variables are presented as mean ± standard deviation (SD) or median [interquartile range (IQR)] and were compared using the Student's *t*-test or Mann–Whitney *U*-test, as appropriate. Categorical variables are expressed as counts (percentages) and compared using the Chi-square test or Fisher's exact test. To identify independent predictors of IAT, univariate logistic regression analysis was first performed. Variables with a *P*-value <0.10 in the univariate analysis, as well as clinically significant parameters regardless of their univariate *P*-value, were entered into the multivariable logistic regression model using a backward stepwise selection method. Odds ratios (ORs) and 95% confidence intervals (CIs) were calculated to evaluate the strength of associations. A two-sided *P*-value <0.05 was considered statistically significant. All analyses were performed using SPSS version 26.0 (IBM Corp., Armonk, NY, USA) and R software version 4.2.0. To validate the significance of the multivariable regression results, least absolute shrinkage and selection operator (LASSO) regression was conducted as a sensitivity analysis. LASSO regression with 5-fold cross-validation was used to select the most relevant predictors, with the area under the curve (AUC) as the optimization criterion for the penalty parameter *λ*. Variables identified by both methods were considered as significant independent risk factors. For patients meeting the criteria for recurrence, Kaplan–Meier analysis was performed to evaluate the impact of IAT on the success rate of AF ablation. A two-tailed *P* value <0.05 was considered statistically significant.

## 3 Results

### 3.1 Patient characteristics and procedure characteristics

**Figure 1:**
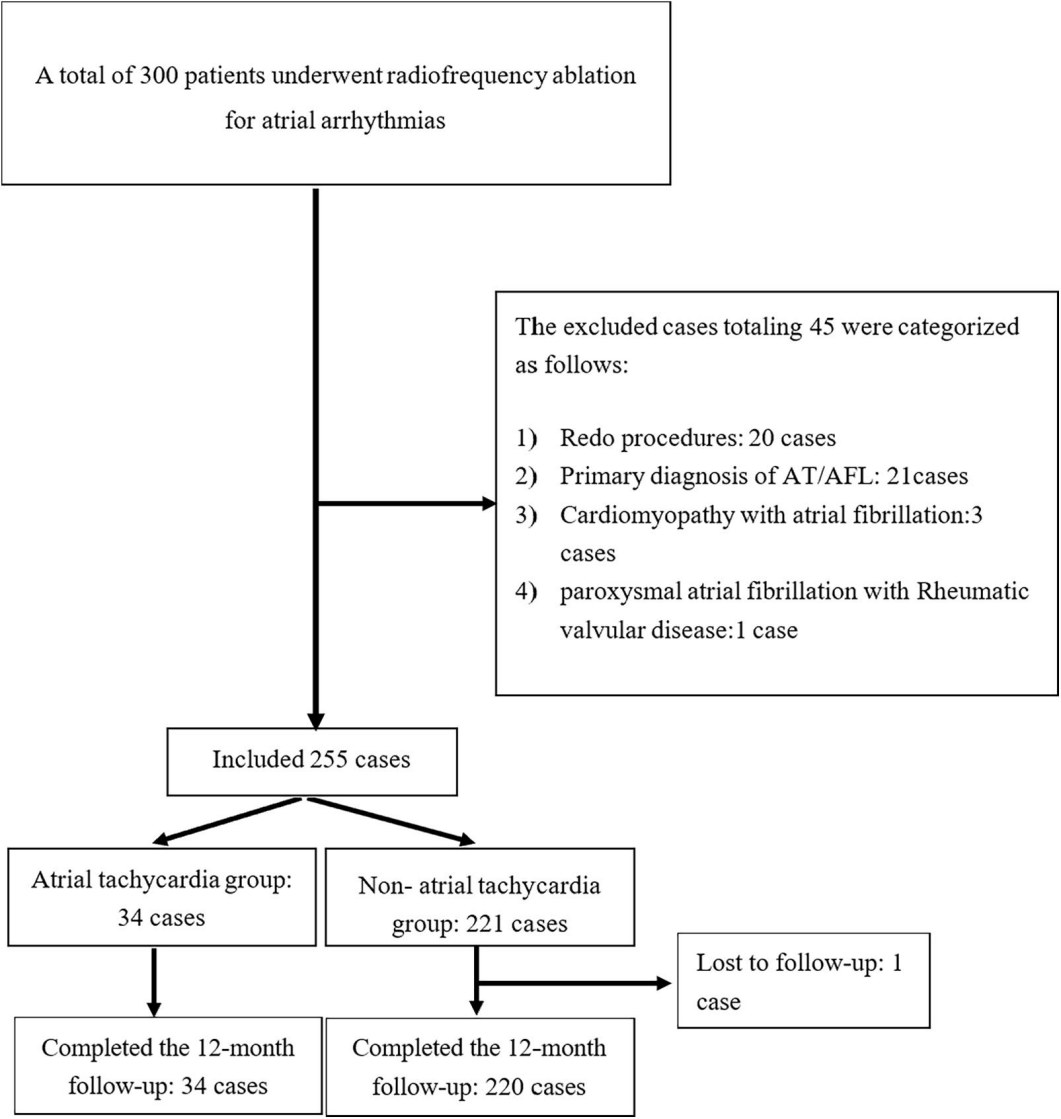
The flow diagram of study.

A total of 300 PAF patients underwent RFCA. Of these, 45 patients were excluded, thus, 255 patients were listed in the final analysis. Among them, 34 (13.33%) patients exhibited stable AT lasting >2 min, either spontaneously or induced during the procedure (Figure 1). No significant differences existed between the two groups regarding age, sex, body mass index, or histories of smoking and alcohol consumption. Significant differences were also absent in comorbidities such as stroke, coronary artery disease, diabetes mellitus, thyroid disorders, or congestive heart failure. However, hypertension prevalence was significantly lower in the AT group compared to the non-AT group (29.41% vs. 47.51%; *P* = 0.048). Most patients received antiarrhythmic medications, including *β*-blockers, prior to the procedure, with no significant intergroup differences. The AT group had a significantly larger mean right atrial diameter (RAD) (38.32 ± 3.88 mm vs. 35.30 ± 4.25 mm; *P* = 0.022). Similarly, patients with AT demonstrated a significantly enlarged LAD measured via transthoracic echocardiography in the parasternal long-axis view during end-systole compared to those without AT (40.09 ± 4.14 mm vs. 38.49 ± 3.69 mm; *P* = 0.022). Left ventricular end-diastolic diameter (LVEDD) was significantly larger in the AT group (49.09 ± 4.56 mm vs. 47.33 ± 3.98 mm; *P* = 0.02), while left ventricular ejection fraction (LVEF) was lower in the AT group but did not reach statistical significance (60.13 ± 7.35% vs. 62.39 ± 4.71%; *P* = 0.09). NT-ProBNP levels, mean CHA₂DS₂-VASc score, and mean HAS-BLED score showed no significant differences (Table 1).

**Table 1:** The baseline characteristics of the intraprocedural AT group and Non-AT group.

Parameters	Total (*n* = 255)	Non-AT group (*n* = 221)	AT group (*n* = 34)	*P* value
Age, (year)	60.02 ± 10.20	60.30 ± 9.83	58.15 ± 12.37	0.25
Female, *n* (%)	94 (36.86)	86 (38.91)	8 (23.53)	0.08
BMI, (Kg/m^2^)	25.09 ± 2.89	25.20 ± 2.98	24.39 ± 2.20	0.13
History, *n* (%)				
Smoking	59 (23.24)	53 (23.98)	6 (8.82)	0.82
Drinking	42 (16.47)	36 (16.29)	6 (17.65)	0.84
Comorbidities, *n* (%)				
Stroke	20 (7.84)	15 (6.79)	5 (14.71)	0.21
CAD	36 (14.12)	32 (14.48)	4 (11.76)	0.87
Hypertension	115 (45.10)	105 (47.51)	10 (29.41)	0.05
Diabetes	33 (12.94)	29 (13.12)	4 (11.76)	1.00
Thyroid disorders	10 (3.92)	10 (4.52)	0 (0.00)	0.43
Heart failure	12 (4.71)	8 (3.62)	4 (11.76)	0.10
AAD, *n* (%)				
Amiodarone	11 (4.31)	7 (3.17)	4 (11.76)	0.07
Propafenone	15 (5.88)	14 (6.33)	1 (2.94)	0.70
*β*-Blockers	93 (36.47)	78 (35.29)	15 (44.12)	0.32
CCB	52 (20.39)	49 (22.17)	3 (8.82)	0.07
LAD, (mm)	38.71 ± 3.78	38.49 ± 3.69	40.09 ± 4.14	0.02
RAD, (mm)	35.71 ± 4.32	35.30 ± 4.25	38.32 ± 3.88	<.001
LVDd, (mm)	47.56 ± 4.10	47.33 ± 3.98	49.09 ± 4.56	0.02
LVEF, (%)	62.09 ± 5.18	62.39 ± 4.71	60.13 ± 7.35	0.09
HAS-BLED	1.42 ± 0.94	1.45 ± 0.96	1.24 ± 0.85	0.22
CHA_2_DS_2_^_^VASc	1.62 ± 1.41	1.63 ± 1.41	1.50 ± 1.44	0.61
NT-proBNP, (pg/ml)	90.32 (45.52, 215.10)	83.49 (46.48, 212.45)	119.70 (40.51, 273.70)	0.48

AT, atrial tachycardia; CAD, coronary artery disease; CCB, calcium channel blocker; LAD, left atrial diameter; LVDd, left ventricular diastolic diameter; LVEF, left ventricular ejection fraction; NT-proBNP, N-terminal pro-brain natriuretic peptide; RAD, right atrial diameter.

Regarding intra-procedural parameters, the AT group demonstrated significantly longer procedure time (215.94 ± 58.74 min vs. 172.33 ± 38.68 min; *P* < 0.001), larger right atrial volumes (87.30 ± 28.52 ml vs. 74.61 ± 23.31 ml; *P* = 0.007), and larger left atrial volumes (131.19 ± 41.43 ml vs. 112.61 ± 27.82 ml; *P* = 0.02) compared to the non-AT group. Fluoroscopy time, fluoroscopy dose, and activated clotting time (ACT) showed no significant differences. Additionally, the number of left and right atrial low voltage zones showed no significant intergroup differences. The AT group required significantly more tricuspid isthmus, mitral isthmus, and coronary sinus (CS) ablations than the non-AT group, whereas no significant differences were found in other linear ablation parameters. No significant differences were observed in peri-procedural complications between the two groups (Table 2).

**Table 2:** The procedural parameters of the intraprocedural AT group and Non-AT group.

Parameters	Total (*n* = 255)	Non-AT group (*n* = 221)	AT group (*n* = 34)	*P* value
Procedure duration, (min)	177.98 ± 44.18	172.33 ± 38.68	215.94 ± 58.74	<.001
x-ray time, (min)	6.40 (4.50, 9.28)	6.51 (4.39, 9.17)	6.13 (5.20, 9.31)	0.50
x-ray dose, (mGy)	9.00 (5.00, 21.00)	10.00 (5.00, 20.50)	8.00 (6.00, 25.00)	0.49
ACT, (s)	271.53 ± 27.39	270.67 ± 27.43	277.48 ± 26.78	0.20
Left atrial volume, (ml)	115.07 ± 30.53	112.61 ± 27.82	131.19 ± 41.43	0.02
Right atrial volume, (ml)	76.28 ± 24.37	74.61 ± 23.31	87.30 ± 28.52	0.007
Number of patients with LLAV, *n* (%)	26 (10.24)	21 (9.50)	5 (15.15)	0.49
Number of patients with RLAV, *n* (%)	1 (0.39)	1 (0.45)	0	1.00
Status of linear ablation, *n* (%)				
Roof lines				0.07
No ablation	139 (54.51)	118 (53.39)	21 (61.76)	
Bidirectional block	115 (45.10)	103 (46.61)	12 (35.29)	
Incomplete block	1 (0.39)	0	1 (2.94)	
Cavotricuspid isthmus lines				0.17
No ablation	210 (82.35)	178 (80.54)	32 (94.12)	
Bidirectional block	41 (16.07)	40 (18.10)	1 (2.94)	
Incomplete block	4 (1.57)	3 (1.36)	1 (2.94)	
Mitral isthmus lines				0.17
No ablation	251 (98.43)	218 (98.64)	33 (97.06)	
Bidirectional block	3 (1.18)	3 (1.36)	0	
Incomplete block	1 (0.39)	0	1 (2.94)	
Anterior wall lines				0.16
No ablation	248 (97.25)	215 (97.29)	33 (97.06)	
Bidirectional block	6 (2.35)	6 (2.71)	0	
Incomplete block	1 (0.39)	0	1 (2.94)	
Overall ablation strategies, *n* (%)				
SVC isolation	233 (91.37)	204 (92.31)	29 (85.29)	0.30
RAA ablation	12 (4.71)	11 (4.98)	1 (2.94)	0.93
CS ablation	18 (7.06)	12 (5.43)	6 (17.65)	0.03
CTI ablation	68 (26.67)	43 (19.46)	25 (73.53)	<.001
Roof line ablation	122 (47.84)	103 (46.61)	19 (55.88)	0.31
Posterior wall isolation	7 (2.75)	4 (1.81)	3 (8.82)	0.05
MI ablation	11 (4.31)	3 (1.36)	8 (23.53)	<.001
Anterior Wall Line ablation	10 (3.92)	6 (2.71)	4 (11.76)	0.04
Perioperative Complications, *n* (%)				
Vascular complications	14 (5.49)	12 (5.43)	2 (5.88)	0.95
Cardiac tamponade	0	0	0	–
Death	0	0	0	–
Sinus arrest/Third-degree AV block	0	0	0	–

ACT, activated clotting time; AT, atrial tachycardia; AV, Atrioventricular; CS, coronary sinus; CTI, cavotricuspid isthmus; LLAV, left atrial low-voltage area; MI, mitral isthmus; RLAV, right atrial low-voltage area; SVC, superior vena cava; RAA, right atrial appendage.

### 3.2 Characteristics of atrial tachycardia

**Figure 2:**
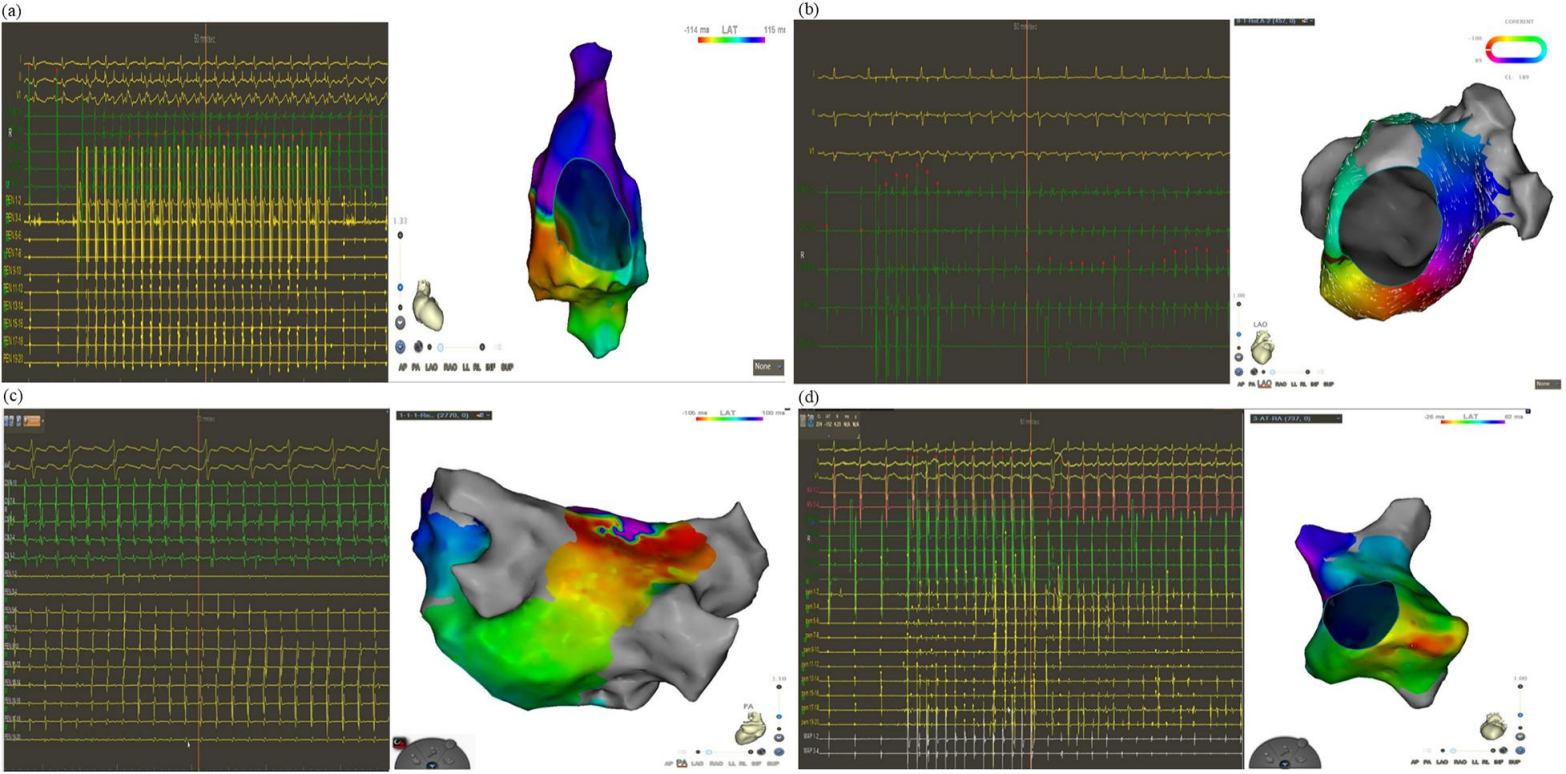
Burst pacing was performed via the coronary sinus or high right atrium at cycle lengths of 200–250 ms after CPVI to assess inducibility of atrial tachyarrhythmias induced peri-tricuspid **(a)**, peri-mitral **(b)**, roof-dependent reentry **(c)**, and focal **(d)** ATs. CPVI, circumferential pulmonary vein isolation.

Among the 34 patients, a total of 40 distinct ATs were identified, with an average cycle length of 235.71 ± 8.04 ms. Electrophysiological features were definitively mapped for each AT. During the procedure, 27.5% of ATs occurred spontaneously, and 65.0% were induced by electrophysiological stimulation. The remaining 5.0% and 2.5% were induced by mechanical and pharmacological stimuli, respectively. Macro-reentrant ATs were the predominant type. The distribution of macro-reentrant AT mechanisms showed dual dominance: 45% were peri-mitral reentry and 45% were peri-tricuspid AT. Roof-dependent ATs were rare, comprising only 2.5%. Focal ATs, representing non-reentrant mechanisms, were observed in 7.5% of ATs during procedures (Table 3) (Figure 2).

**Table 3:** Electrophysiological characteristics of intraprocedural AT.

Electrophysiological characteristics	Value
Total, *n* (%)	255
Number of patients with AT, *n* (%)	34 (13.33)
Number of AT episodes, *n* (%)	40
Mapping points, *n* (25%, 75%)	967 (50, 1,435)
AT cycle length (ms)	235.71 ± 8.04
Induction conditions of AT, *n* (%)	
Programmed stimulation	26 (65.0)
Spontaneous	11 (27.5)
Mechanical stimulation	2 (5.0)
Pharmacological induction	1 (2.5)
AT mechanism, *n* (%)	
Peri-mitral	18 (45)
Peri-tricuspid	18 (45)
Roof-dependent	1 (2.5)

AT, atrial tachycardia.

### 3.3 Risk factors for intra-procedural atrial tachycardia

Non-collinear variables with a univariate analysis *P* value <0.1 including LVEF, RAD, LAD, LVEDD, sex, and hypertension—were included in the binary logistic regression analysis. The results indicated that increased RAD was the only independent risk factor for IAT (*P* = 0.015), with an odds ratio (OR) of 1.14 (95% CI: 1.026–1.26) (Table 4).

**Table 4:** Univariate and multivariate regression analysis of risk factors for intraprocedural AT.

	Univariate analysis				Multivariate analysis			
	β	S.E	*P*	OR (95%CI)	β	S.E	*P*	OR (95%CI)
Sex								
Female				1.00 (Reference)				1.00 (Reference)
Male	0.73	0.43	0.088	2.07 (0.90–4.78)	0.41	0.47	0.382	1.51 (0.60–3.77)
Hypertension								
No				1.00 (Reference)				1.00 (Reference)
Yes	−0.78	0.40	0.052	0.46 (0.21–1.01)	−0.75	0.42	0.074	0.47 (0.21–1.08)
LAD	0.12	0.05	0.023	1.12 (1.02–1.24)	0.03	0.06	0.655	1.03 (0.91–1.16)
RAD	0.16	0.04	<.001	1.17 (1.08–1.28)	0.13	0.05	0.015	1.14 (1.02–1.26)
LVDd	0.11	0.05	0.020	1.11 (1.02–1.22)	0.03	0.05	0.564	1.03 (0.93–1.14)
LVEF	−0.06	0.03	0.026	0.94 (0.89–0.99)	−0.03	0.03	0.339	0.97 (0.91–1.03)

AT, atrial tachycardia; CI, confidence interval; LAD, left atrial diameter; LVDd, left ventricular diastolic diameter; LVEF, left ventricular ejection fraction; OR, odds ratio; RAD, right atrial diameter.

### 3.4 LASSO regression for variable selection

Due to the limited number of positive events (*n* = 34), LASSO regression was employed for variable selection to avoid over fitting. Using 5-fold cross-validation with the AUC as the optimization criterion, *λ*.1se (the largest *λ* within one standard error of the minimum cross-validation error) was selected to construct the most parsimonious model. LASSO regression revealed that among all potential predictors, only RAD was retained as a non-zero coefficient variable associated with IAT in PAF, with a LASSO coefficient of 0.039. The coefficients of other variables were shrunk to zero, suggesting that these variables had weak or unstable associations with the IAT (Supplementary Figures S1, S2).

ROC curve analysis of the multivariable logistic regression model yielded an AUC of 0.71 (95% CI: 0.62–0.79), indicating moderate discriminative ability, corresponding to a sensitivity of 65% and a specificity of 70% (Supplementary Figure S3). Both traditional multivariable logistic regression and LASSO regression consistently identified RAD as an independent risk factor for IAT. As a penalized regression method that shrinks irrelevant variable coefficients to zero, LASSO regression further validated the significance and reliability of the multivariable regression findings.

### 3.5 Follow-up

**Figure 3:**
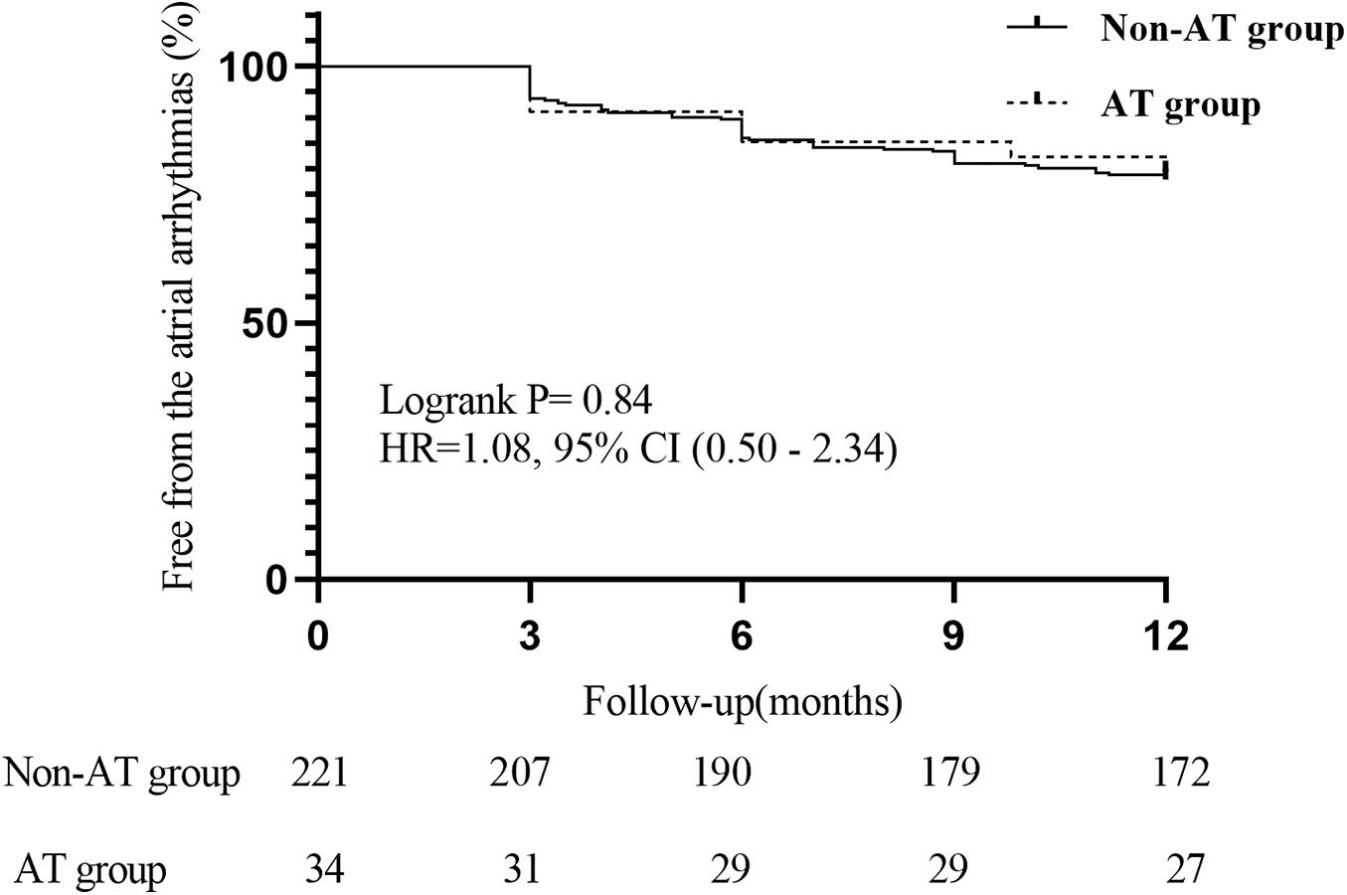
The kaplan–meier curve illustrates event-free survival rates between patients with and without intra-procedural atrial tachycardia.

During the 12-months post-ablation follow-up, 1 patient in the non-IAT group was lost to follow-up. Consequently, a total of 254 patients (220 in the non-IAT group and 34 in the IAT group) were included in the final outcome analysis. Arrhythmia recurrence occurred in 49 of 220 patients (22.3%) in the non-IAT group and 7 of 34 patients (20.6%) in the IAT group. Following the 3-month blanking period and discontinuation of propafenone, most patients with recurrence opted for medical therapy (primarily resuming oral AADs) or observation. Specifically, in the non-IAT group, 43 of 49 patients with recurrence (19.5% of the total group) chose for symptomatic management with AADs or observation, while 6 underwent redo ablation. In the IAT group, 5 of 7 patients with recurrence (14.7% of the total group) chose medical management. There was no significant difference between the two groups in the proportion of patients who required a resumption of AADs during the follow-up period (19.5% vs. 14.7%, *P* = 0.51). The overall 12-month sinus rhythm maintenance rate without antiarrhythmic drug use was approximately 78.0%. Specifically, the non-AT group exhibited a 12-month sinus rhythm maintenance rate of 77.8%, while the AT group demonstrated a rate of 79.4%. Statistical analysis revealed no significant difference in sinus rhythm maintenance rates between the two groups (*P* = 0.84) (Figure 3).

## 4 Discussion

### 4.1 The incidence and forms of IAT

To date, no dedicated studies have specifically reported the incidence of IAT during PAF ablation. Current literature primarily focuses on the incidence and management of post-ablation AT. Historically, IAT incidence has varied widely (10%–60%), largely dependent on the ablation strategies employed (12–14). For instance, Nam et al. reported an AT incidence of 14.9% following CPVI alone, closely aligning with the 13.33% observed in this study. However, electrogram-guided ablation increased this proportion, with 15.7% of patients developing inducible or spontaneous AT (12). Similarly, Nagamoto et al. documented a 27% incidence of IAT in PAF patients, and later reported a 56% incidence among patients with long-standing persistent AF undergoing CPVI plus electrogram-guided ablation (13, 14). These higher rates likely reflect differences in patient populations (including persistent AF) and the application of more extensive ablation strategies.

Reports detailing the specific forms of IAT are limited. Chae et al. noted that over 88% of post-ablation AT cases were reentrant, with 90% associated with gaps in prior ablation lines (15). Chugh identified that 61% of intra-procedural or post-ablation AT cases related to the mitral isthmus (16). In the present study, reentrant AT involving the tricuspid and mitral isthmus accounted for over 60% of identified cases. Although reentrant mechanisms remain the predominant form, the proportion associated with gaps was lower than in Chae's report (15), likely reflecting the less aggressive ablation strategies for PAF used here and the widespread adoption of contact force-sensing catheters. A recent study by Duytschaever et al. explores AT reentry mechanisms and ablation strategies, utilizing topological principles to provide a comprehensive understanding of the underlying processes (17).

### 4.2 The risk factors of IAT

Advancements in understanding post-ablation AT mechanisms have highlighted non-bidirectional conduction block during linear ablation and electrogram-guided ablation as significant risk factors (15, 18, 19). Despite the dominance of CPVI as the primary PAF ablation strategy, spontaneous or inducible IAT occurred in 13.33% of patients here. The underlying mechanisms remain incompletely understood. Prior studies link left and right atrial enlargement, linear ablation, and complex fractionated atrial electrograms (CFAE) ablation to the increased post-ablation AT risk (19–22). Yamada and Veenhuyzen reported that residual gaps in pulmonary vein isolation lines contribute to post-ablation AT (23, 24). In this study, right atrial enlargement emerged as a significant risk factor for IAT, potentially increasing susceptibility to peri-tricuspid AT—a finding consistent with prior research (20). This finding is of significant clinical interest, as conventional electrophysiological paradigms typically emphasize the predominant role of LA remodeling in the pathogenesis and maintenance of AF (25). Several pathophysiological mechanisms may elucidate the superior predictive utility of RAD observed in this cohort: While LAD is an established metric of AF chronicity, RA enlargement may signify a more pervasive disease process that has progressed beyond the PVs. In patients manifesting RA enlargement, the atrial substrate likely exhibits increased heterogeneity—characterized by abbreviated refractoriness and impaired conduction velocity—which facilitates the transition from AF to organized, stable IAT following CPVI (26). The geometric and electrophysiological properties of a dilated RA provide an increased “critical mass” and elongated potential reentrant pathways, inherently stabilizing macro-reentrant circuits (27). This aligns with our observation that a significant proportion of IATs were RA-dependent. Although the LAD was larger in the AT group than the non-AT group, the difference lacked statistical significance in multivariate regression analysis, possibly due to limited sample size. Furthermore, as this study exclusively included PAF cases, linear ablations other than roof line were rarely performed, resulting in no significant difference in incomplete linear block rates between groups.

### 4.3 Clinical implications of IAT

The optimal clinical management of IAT remains debated. Aman Chugh reported that 55% of patients with IAT experienced recurrence during follow-up, whereas 33% of post-ablation AT cases resolved spontaneously within five months (16). Yu et al. proposed electrical cardioversion as a viable strategy for patients with peri-mitral reentrant AT and well-preserved left atrial substrate, followed by monitoring during follow-up (28). Moreover, randomized controlled trials demonstrate that ablating IAT improves long-term sinus rhythm maintenance compared to cardioversion alone (29). In this study, the one-year sinus rhythm maintenance rate showed no significant difference between patients with treated IAT and those without AT, consistent with findings by Nagamoto et al. (14) and LEONG-SIT *P* et al. (6). It is important to note that the AT group required significantly longer procedure times and more extensive linear ablations (Table 2). This suggests that the comparable 12-month success rate was likely achieved through the systematic elimination of these intra-procedural arrhythmias. Therefore, our findings underscore the clinical importance of mapping and ablating IAT when it occurs, rather than suggesting that its occurrence is of no prognostic significance.

### 4.4 Limitations

This study has two primary limitations. First, its single-center observational design may limit the generalization of the results. Differences in patient populations, technical approaches, and treatment strategies across institutions or regions could affect the applicability of these findings elsewhere. Second, although 255 patients met the inclusion criteria, the sample size of the AT group remains relatively small (*n* = 34). This limited number of patients and subsequent recurrence events may constrain the study's statistical power, making it difficult to definitively exclude a clinically meaningful difference in long-term sinus rhythm maintenance rate between the two groups. Therefore, our finding of comparable 12-month outcomes should be interpreted with caution, and larger multicenter studies are needed to support these results.

## 5 Conclusion

IAT occurs in approximately 13.33% of patients undergoing PAF ablation procedures, predominantly manifesting as reentry involving the peri-tricuspid and peri-mitral regions. Right atrial enlargement is a key risk factor. Critically, with successful intra-procedural identification and proper management within the limitations of our study's sample size, patients with appropriately managed IAT showed a 12-month success rate comparable to those without IAT. However, larger multicenter studies are required to confirm whether a subtle but clinically meaningful difference in long-term prognosis exists, underscoring the importance of its timely intra-procedural identification and management. These findings provide valuable insights into the mechanisms, management, and clinical outcomes of IAT in PAF ablation.

## Data Availability

The original contributions presented in the study are included in the article/Supplementary Material, further inquiries can be directed to the corresponding author/s.
