# Supplementary material for: Intraprocedural atrial tachycardia during ablation of paroxysmal atrial fibrillation: incidence, mechanisms, and clinical outcomes
Source: Front Cardiovasc Med. 2026 Apr 22;13:1822766. doi: 10.3389/fcvm.2026.1822766 (PMC13143780; doi:10.3389/fcvm.2026.1822766)
Supplement: Supplementary file 1 [file Table1.docx]

**Figure S1** LASSO cross-validation curve. The plot shows the 5-fold cross-validation AUC as a function of log(λ). The left vertical dashed line (blue) indicates λ.min, and the right vertical dashed line (red) indicates λ.1se. The numbers at the top represent the number of non-zero coefficients at each λ value. LASSO, least absolute shrinkage and selection operator.

**Figure S2** LASSO coefficient path plot. Each colored line represents the trajectory of a variable's coefficient as λ varies. Vertical dashed lines indicate λ.min (blue) and λ.1se (red). Variables with non-zero coefficients at λ.1se were retained in the final model. LASSO, least absolute shrinkage and selection operator.

**Figure S3** Receiver operating characteristic (ROC) curve of the LASSO-derived model for predicting IAT. The blue line represents the predictive performance of the model (AUC = 0.705, 95% CI: 0.615–0.794). The diagonal grey line represents the reference line (AUC = 0.5), indicating the performance of a random classifier. AUC, area under the curve; CI, confidence interval; IAT, intraprocedural atrial tachycardia..
