# Supplementary figures and images for: Intraprocedural atrial tachycardia during ablation of paroxysmal atrial fibrillation: incidence, mechanisms, and clinical outcomes
Source: Front Cardiovasc Med. 2026 Apr 22;13:1822766. doi: 10.3389/fcvm.2026.1822766 (PMC13143780; doi:10.3389/fcvm.2026.1822766)

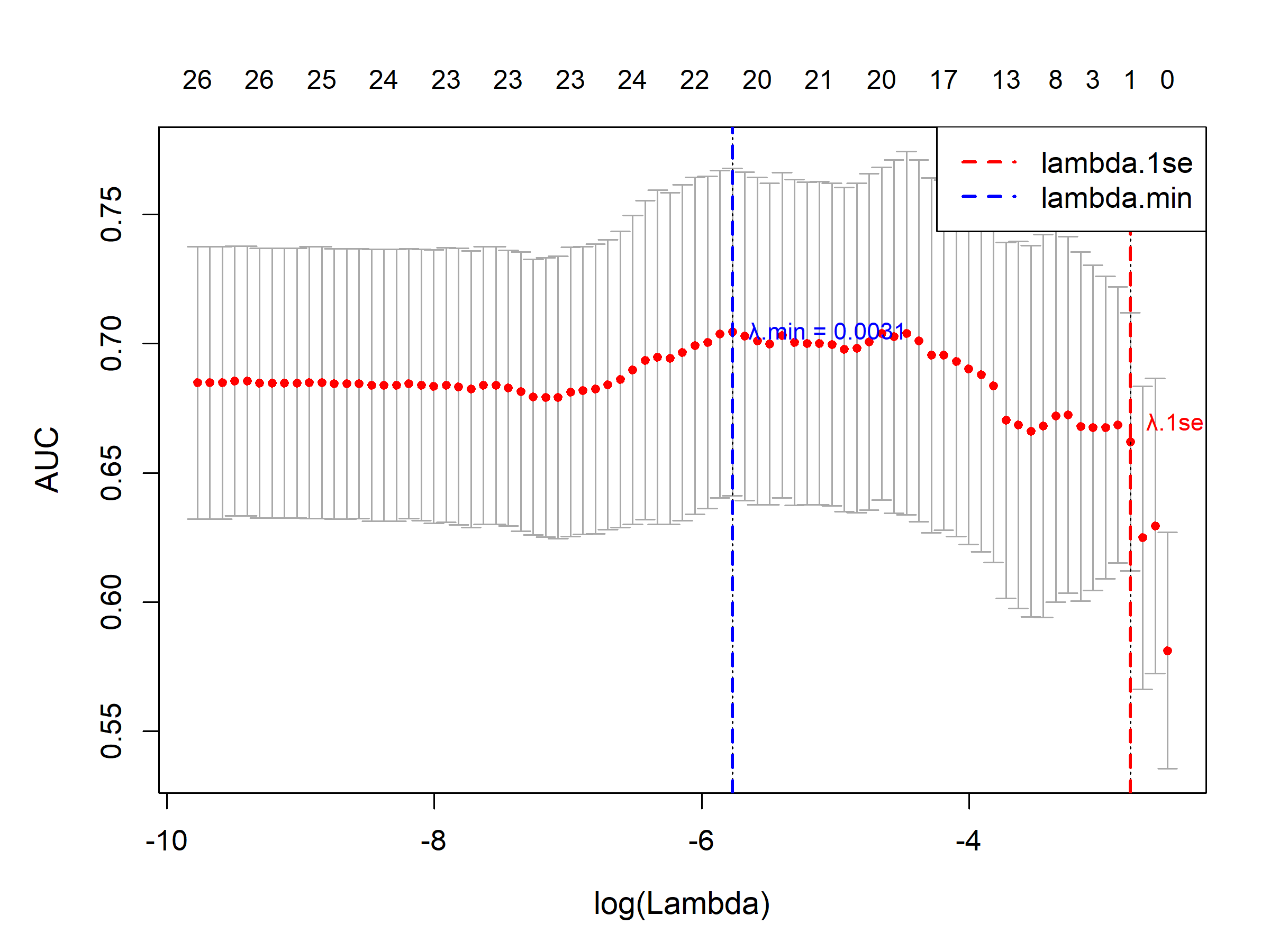

Supplement: Supplementary file 2 [file Image1.tiff]

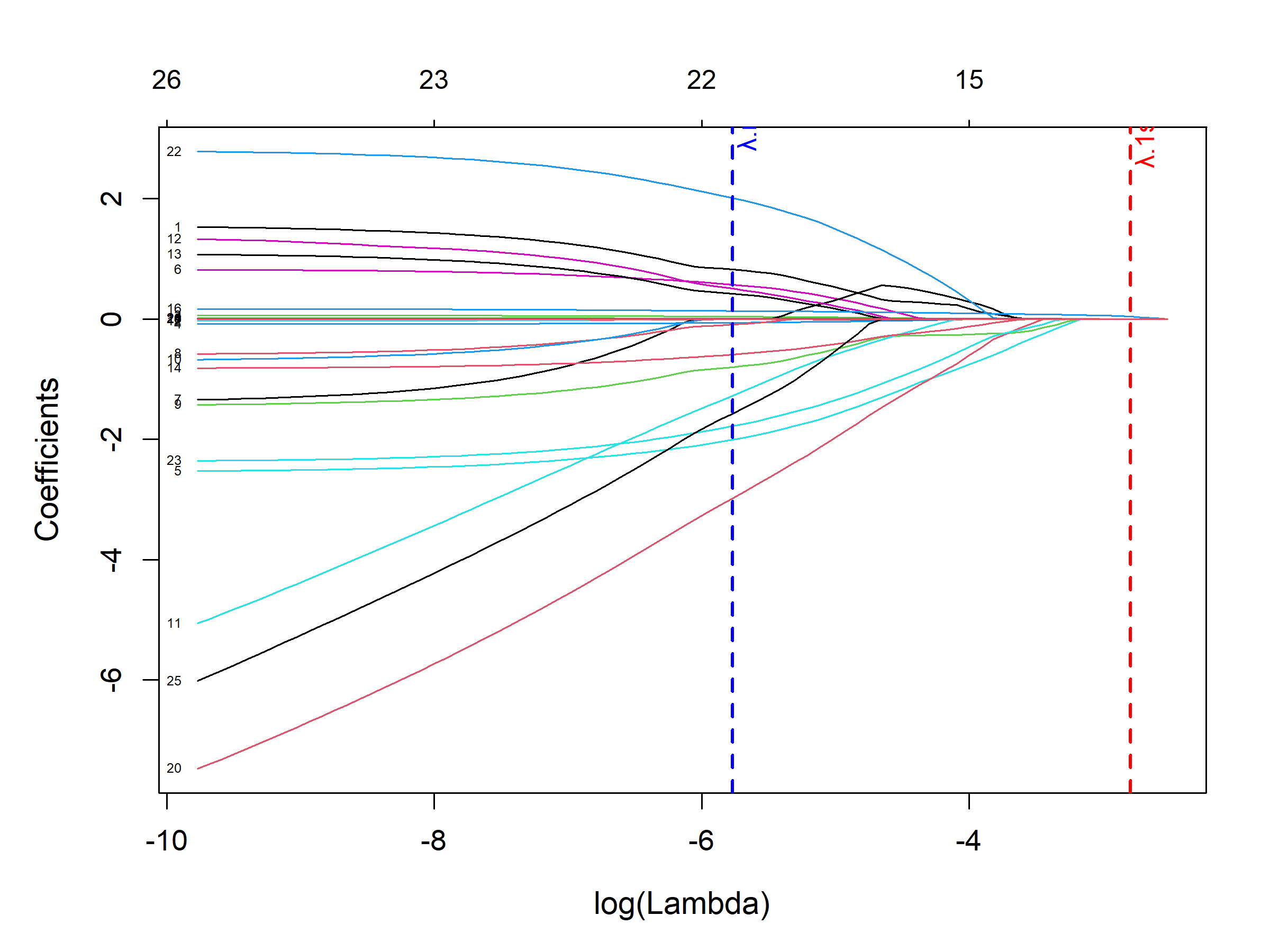

Supplement: Supplementary file 3 [file Image2.tiff]

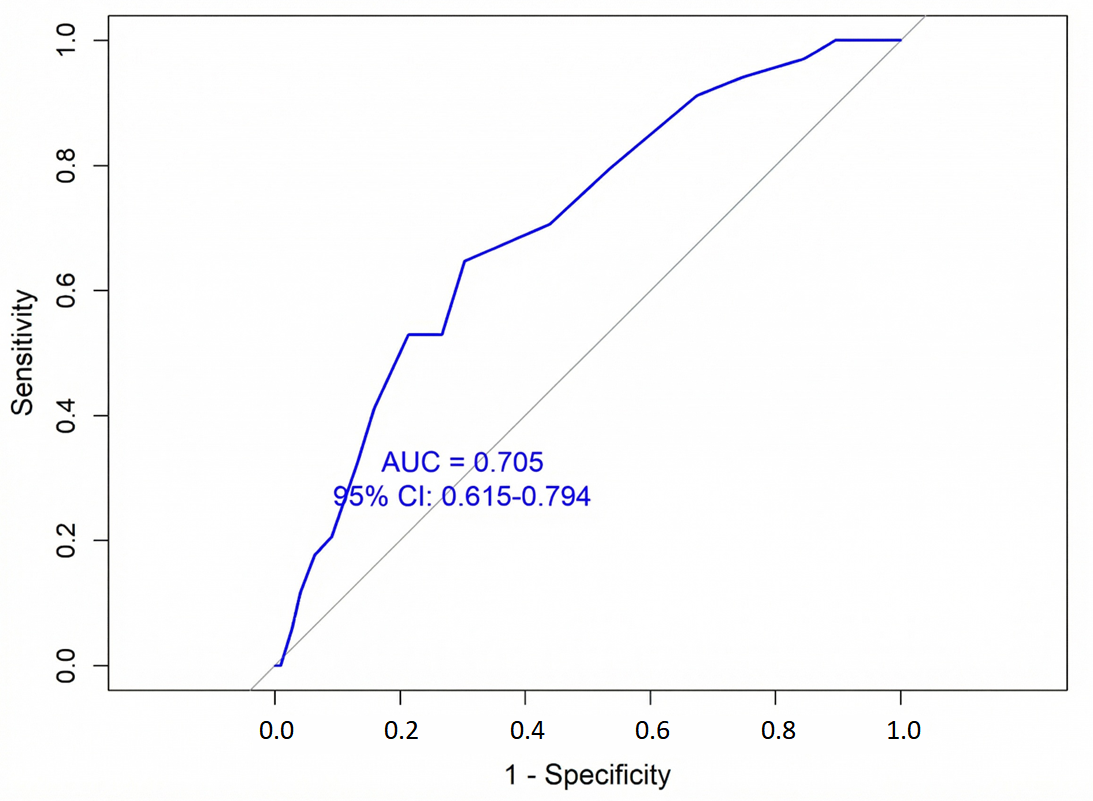

Supplement: Supplementary file 4 [file Image3.tif]
